# Functional hepatic deterioration determined by ^13^C-methacetin breath test is associated with impaired hemodynamics and late Fontan failure in adults

**DOI:** 10.3389/fcvm.2022.952080

**Published:** 2022-09-07

**Authors:** Anastasia Schleiger, Peter Kramer, Hannes Sallmon, Niklas Jentsch, Marta Pileckaite, Friederike Danne, Marie Schafstedde, Hans-Peter Müller, Tobias Müller, Frank Tacke, Maximilian Jara, Martin Stockmann, Felix Berger, Stanislav Ovroutski

**Affiliations:** ^1^Department of Congenital Heart Disease/Pediatric Cardiology, Deutsches Herzzentrum Berlin, Berlin, Germany; ^2^Institute for Cardiovascular Computer-Assisted Medicine, Charité—Universitätsmedizin Berlin, Berlin, Germany; ^3^Berlin Institute of Health, Charité-Universitätsmedizin Berlin, Berlin, Germany; ^4^Charité Centre for Internal Medicine and Dermatology, Charité-Universitätsmedizin Berlin, Berlin, Germany; ^5^Department of Gastroenterology and Hepatology, Charité—Universitätsmedizin Berlin, Berlin, Germany; ^6^Department of General, Visceral and Vascular Surgery, Charité—Universitätsmedizin Berlin, Berlin, Germany; ^7^Department of Pediatric Cardiology, Charité—Universitätsmedizin Berlin, Berlin, Germany

**Keywords:** late Fontan failure, Fontan-associated liver disease, end-organ dysfunction, Fontan hemodynamics, metabolic liver function

## Abstract

**Background:**

Despite improved survival a substantial number of Fontan patients eventually develop late failure. Fontan-associated liver disease (FALD) is the most frequent end-organ dysfunction. Although impaired hemodynamics and Fontan failure correlate with FALD severity, no association between hepatic functional metabolic impairment and Fontan hemodynamics has been established.

**Hypothesis:**

Metabolic liver function measured by liver maximum function capacity test (LiMAx®) correlates with Fontan hemodynamics and Fontan failure.

**Methods:**

From 2020 to 2022, 58 adult Fontan patients [median age: 29.3 years, IQR (12.7), median follow-up time after Fontan operation: 23.2 years, IQR (8.7)] were analyzed in a cross-sectional study. Hemodynamic assessment included echocardiography, cardiopulmonary exercise testing and invasive hemodynamic evaluation. Fontan failure was defined based on commonly applied clinical criteria and our recently composed multimodal Fontan failure score.

**Results:**

LiMAx® test revealed normal maximum liver function capacity in 40 patients (>315 μg/h^*^kg). In 18 patients a mild to moderate impairment was detected (140–314 μg/h^*^kg), no patient suffered from severe hepatic deterioration (≤ 139 μg/kg^*^h). Fontan failure was present in 15 patients. Metabolic liver function was significantly reduced in patients with increased pulmonary artery pressure (*p* = 0.041. r = −0.269) and ventricular end-diastolic pressure (*p* = 0.033, r = −0.325), respectively. In addition, maximum liver function capacity was significantly impaired in patients with late Fontan failure (289.0 ± 99.6 μg/kg^*^h vs. 384.5 ± 128.6 μg/kg^*^h, *p* = 0.007).

**Conclusion:**

Maximum liver function capacity as determined by LiMAx® was significantly reduced in patients with late Fontan failure. In addition, elevated pulmonary artery pressure and end-diastolic ventricular pressure were associated with hepatic functional metabolic impairment.

## Introduction

Despite its tremendous success in treating patients with univentricular anatomy, the Fontan operation remains a palliative procedure, which is characterized by abnormal hemodynamics (1, 2). In the long-term course, chronic venous congestion and low cardiac output lead to progressive clinical heart failure with limited treatment options (3, 4). The relative scarceness of effective pharmacological therapies and the limited applicability of mechanical circulatory support restrict end-stage therapeutic strategies to cardiac transplantation, which itself is associated with considerable morbidity and mortality (5, 6). The indications and optimal timing of cardiac transplantation in Fontan patients are still subject of ongoing debate. The urgency of addressing these issues is illustrated by the fact that within the next decades a significant increase in adult Fontan patients experiencing hemodynamic compromise and subsequently cardio-circulatory demise can be expected (7).

Liver-associated morbidity and mortality are well described in the adult Fontan population and constitute major risk factors significantly impacting survival rates after cardiac transplantation (8, 9). Additionally, the indication for a combined heart and liver transplantation is currently subject of ongoing debate. Therefore, reliable diagnostic modalities are required to monitor hepatic end-organ damage, determine the optimal timing for cardiac transplantation and define the indications for a combined heart-liver transplantation. The liver maximum capacity test (LiMAx®) has been developed to quantitatively determine metabolic liver function. Methacetin is exclusively metabolized by the cytochrome P4501A2 (CYP1A2) system, which exclusively exists in hepatocytes (10). Therefore, enzymatic cleavage of intravenously administrated ^13^C-methacetin into ^13^CO_2_, reliably correlates with hepatic parenchymal volume and metabolic function (10). Previously, we have demonstrated that structural hepatic alterations antecede functional hepatic impairment as assessed by LiMAx®, with maximum liver function capacity being well preserved in the majority of Fontan patients (11). However, the potential impacts of late Fontan failure and hemodynamics on metabolic liver function are unknown.

Herein, we aimed to analyze the potential associations between maximum liver function capacity and (I) Fontan hemodynamics by clinical, echocardiographic and invasive assessments and (II) late Fontan failure.

## Methods

### Study design and patients

From 2019 to 2022 58 adult Fontan patients, who successively presented in our outpatient clinic for follow-up, were included in our cross-sectional observational study. All patients received measurement of maximal liver function capacity using LiMAx® test as well as a detailed hemodynamic and hepatic assessment. Exclusion criteria were intolerance to methacetin or paracetamol and/or patient age below 18 years. The institutional review board and ethics committee approved the study (decision number: EA2/127/18). All individual participants consented to participate in the study prior to inclusion.

### Hemodynamic assessment

Hemodynamic assessment included clinical evaluation, echocardiography, cardiopulmonary exercise testing (CPET) and cardiac catheterization. Systolic ventricular function was measured by echocardiography based on the modified Simpson's method (12). Atrioventricular valve incompetence (AVVI) was classified as absent/mild, moderate or severe by visual assessment of the regurgitation jet dimensions in color Doppler sonography. CPET was performed following a standardized institutional protocol using a cycle ergometer. Peak oxygen uptake (VO_2_peak) was measured in ml/kg^*^min and normalized in % of age-, gender- and body dimension-adjusted reference values. Cardiac catheterization included measurements of mean pulmonary artery pressure (mPAP) and systemic ventricular end-diastolic pressure (SVEDP). Transpulmonary pressure gradient (TPG) was calculated as the difference between mPAP and pulmonary capillary wedge pressure. Cardiac output (CO) and pulmonary vascular resistance (PVR) were determined by Fick's principle using oximetry (13). For comparability, CO and PVR are indexed to body-surface area (Cardiac index, CI, l/min/m^2^; pulmonary vascular resistance index, PVRi, WU^*^m^2^). Fontan failure was defined as severe dysfunction of the Fontan circulation caused by impaired ventricular function, moderate to severe atrioventricular valve incompetence, increased pulmonary vascular resistance, recurrent arrhythmia or therapy-refractory protein-losing enteropathy based on commonly applied clinical criteria (14) and our previously described Fontan failure score (15). Briefly, the score includes a set of 15 clinical, echocardiographic, invasive hemodynamic and laboratory parameters and is calculated by assigning one score point for each score item beyond the defined threshold with a range from 0 to 15 points. A score ≥ 8 score points detects late Fontan failure with a sensitivity of 99.3 % and a specificity of 53.9 % (15).

### Hepatic assessment

Hepatic assessment was performed based on our previously published institutional protocol (16) and consisted of laboratory analyses, hepatic ultrasound and liver stiffness measurement by transient elastography (TE). FibroTest® was computed on Biopredictive website (Paris, France; www.biopredictive.com).

### Maximal liver function capacity

The LiMAx® test was performed following the standardized protocol of Stockmann et al. (10).

Briefly, a body weight-adjusted solution (2 mg/kg) of ^13^C-labeled methacetin was administered intravenously. The hepatozyte-specific CYP1A2 system metabolizes ^13^C-labeled methacetin into paracetamol and ^13^CO_2_, which is continuously measured in the exhaled air. The LiMAx test result is calculated based on the individually determined maximum delta-over-baseline ratio of ^13^CO_2_/^12^CO_2_ (10).

## Statistical analysis

Data were collected from medical records of the German Heart Centre Berlin. Data are expressed as median and interquartile range, which were calculated as the 75th minus 25th percentile. Fontan follow-up duration was defined as the interval between Fontan operation and last follow-up. Correlations between maximum liver capacity, echocardiographic, hemodynamic and hepatic parameters as well as the Fontan failure score were assessed using Spearman's correlation and Mann-Whitney U test. Statistical analyses were performed using SPSS statistical software (version 23, IBM Corp., NY, USA). A *p-*value < 0.05 was considered statistically significant.

## Results

### Patient cohort

Patient characteristics of the entire cohort are provided in Table 1. Median patient age was 29.3 years (IQR 12.7) and median follow-up time after Fontan operation 23.2 (IQR 8.7). The most common underlying cardiac morphologies were tricuspid atresia (n = 18), double inlet left ventricle (n = 15) and unbalanced atrioventricular septal defect (n = 9). Fontan modifications consisted of extracardiac conduit in 22 patients, lateral tunnel in 17 patients and atriopulmonary/ atrioventricular connection (APC/AVC) in 19 patients. From the study cohort, 3 patients died during follow-up; 2 patients after cardiac transplantation and 1 patient on mechanical circulatory support. Two additional patients successfully underwent cardiac transplantation.

**Table 1:** Patient characteristics.

	**Entire cohort**	**No Fontan failure**	**Fontan failure**	***P*-value**
	**(n = 58)**	**(n = 43)**	**(n = 15)**	
Patient age (years)	29.3 (12.7)	27.8 (10.9)	36.3 (22.0)	0.093
Age at Fontan operation (years)	5.7 (9.0)	4.6 (8.3)	11.4 (7.8)	**0.035**
Follow-up after Fontan (years)	23.2 (8.7)	23.0 (9.1)	24.6 (11.1)	0.214
Cardiac anatomy (n)				0.541
Tricuspid atresia	18 (31.0%)	14 (32.6%)	4 (26.7%)	
Double inlet left ventricle	15 (25.9%)	11 (26.2%)	4 (26.7%)	
Hypoplastic left heart syndrome	2 (3.5%)	1 (2.3%)	1 (6.7%)	
Complex TGA	3 (5.1%)	2 (4.7%)	1 (6.7%)	
Unbalanced AVSD	9 (15.5%)	5 (11.6%)	4 (26.7%)	
Other	11 (18.9%)	9 (20.9%)	2 (13.3%)	
Left ventricular morphology (n)	39 (67.2%)	31 (72.1%)	8 (53.3%)	0.213
Fontan type (n)				0.373
Extracardiac conduit	22 (37.9%)	16 (37.2%)	6 (40.0%)	
Intracardiac lateral tunnel	17 (29.3%)	12 (27.9%)	5 (33.3%)	
APC / AVC	19 (32.8%)	15 (34.9%)	4 (26.7%)	

Data are presented as median and interquartile range or frequencies and %. Statistically significant results are given in bold letters. APC, atriopulmonary connection; AVC, atrioventricular connection; AVSD, atrioventricular septal defect; TGA, transposition of the great arteries.

### Hemodynamic assessment

Results of hemodynamic assessment are presented in Table 2. Systolic ventricular function was preserved/ mildly impaired in 47 patients, moderately impaired in 9 patients and severely impaired in 2 patients. AVVI was classified as absent/mild in 44 patients, moderate in 13 patients and severe in 1 patient. Median percentage of reference VO_2_peak was 44.4 % (IQR 21.3) in the entire cohort. Invasive hemodynamic evaluation revealed mPAP, SVEDP and TPG to be within normal reference ranges (Table 1). Calculated median CI was 2.2 L/min/m^2^ (IQR 0.8) and median PVRi 2.3 WU^*^m^2^ (IQR 1.1). Late Fontan failure was diagnosed in 15 patients.

**Table 2:** Hemodynamic assessment.

	**Entire cohort**	**No Fontan failure**	**Fontan failure**	***P*-value**
	**(n = 58)**	**(n = 43)**	**(n = 15)**	
Ejection fraction (%)	48.0 (11.3)	50.0 (9.0)	40.0 (16.5)	**<0.001**
≤ 45% (n)	18/54 (33.3%)	9/41 (22.0%)	9/13 (69.2%)	**0.005**
AV valve regurgitation (n)				
Absent/mild	44/58 (75.9%)	36/43 (83.7%)	8/15 (53.3%)	0.056
Moderate	13/58 (22.4%)	7/43 (16.3%)	6/15 (40.0%)	
Severe	1/58 (1.7%)	0 (0.0%)	1 (6.7%)	
Cardiopulmonary exercise testing VO_2_peak (% of reference)	44.4 (21.3)	46.0 (24.5)	30.4 (31.4)	**<0.001**
<50% of reference (n)	31/50 (62.0%)	19/37 (51.4%)	12/13 (92.3%)	**0.008**
Transcutaneous oxygen saturation (%)				
at rest	93.0 (4.3)	95.0 (4.0)	92.0 (5.0)	**0.004**
at VO_2_peak	92.0 (9.8)	92.0 (9.0)	88.0 (11.0)	0.222
mPAP (mmHg)	12.0 (6.3)	10.0 (4.0)	17.0 (6.0)	**<0.001**
≥ 15 mmHg (n)	16/58 (27.6%)	5/43 (11.6%)	11/15 (73.0%)	**<0.001**
SVEDP (mmHg)	10.0 (7.0)	9.0 (6.0)	14.5 (4.8)	**<0.001**
≥ 12 mmHg (n)	20/43 (46.5%)	8/29 (27.6%)	12/14 (85.7%)	**0.001**
TPG (mmHg)	3.5 (1.0)	4.0 (1.0)	3.0 (5.0)	0.551
CO (L/min)	3.9 (1.4)	3.9 (1.4)	4.1 (1.8)	0.664
CI (L/min*m^2^)	2.2 (0.8)	2.3 (0.8)	2.1 (0.7)	0.18
PVR (WU)	0.9 (0.6)	0.8 (0.6)	1.1 (0.9)	0.117
PVRi (WU*m^2^)	1.7 (1.1)	1.6 (1.0)	2.0 (1.9)	0.117
≥ 2.5 WU*m^2^ (n)	10/55 (18.2%)	4/40 (10.0%)	6/15 (40.0%)	**0.018**

Data are presented as median and interquartile range or frequencies (%). Statistically significant results are given in bold letters. AV, atrioventricular; CI, cardiac index; CO, cardiac output; mPAP, mean pulmonary artery pressure; PVR, pulmonary vascular resistance; PVRi, pulmonary vascular resistance index; SVEDP, systemic ventricular end-diastolic pressure; TPG, transpulmonary pressure gradient.

### Hepatic assessment

Results of hepatic assessment are listed Table 3. The laboratory parameters Alanin-Aminotransferase (ALT), Aspartat-Aminotransferase (AST), bilirubin and thrombocytes did not significantly differ between patients with and without Fontan failure, whereas γ-glutamyl-transferase (γGT) was significantly increased in patients with a failing Fontan circulation (*p* = 0.017). Results from hepatic ultrasound revealed that surface nodularity, ascites and segmental atrophy/ hypertrophy were more frequently detected in patients with Fontan failure (Table 3). Additionally, liver stiffness values measured by TE were significantly higher in patients with a failing Fontan circulation (*p* = 0.001), whereas Fibrotest® fibrosis score did not differ between patients with and without Fontan failure.

**Table 3:** Hepatic assessment.

	**Entire cohort**	**No Fontan failure**	**Fontan failure**	***P*-value**
	**(n = 58)**	**(n = 43)**	**(n = 15)**	
Laboratory parameters				
ALT (U/l)	30.1 (7.5)	30.6 (7.3)	30.0 (10.0)	0.901
AST (U/l)	31.0 (12.8)	31.4 (12.9)	27.5 (15.7)	0.189
γGT (U/l)	91.8 (60.5)	79.2 (61.0)	119.8 (51.4)	**0.017**
Total bilirubin (mg/dl)	1.0 (0.7)	1.0 (0.7)	1.0 (1.4)	0.195
Thrombocytes (K/μl)	164.5 (69.5)	165.0 (69.0)	155.0 (74.0)	0.683
Fibrotest® Fibrosis Score	0.6 (0.3)	0.6 (0.3)	0.7 (0.3)	0.434
TE (kPa)	19.4 (19.2)	16.2 (13.0)	33.7 (21.0)	**0.001**
Hepatic ultrasound findings (n)				
Hepatomegaly	11/54 (20.4 %)	8/40 (20.0 %)	3/14 (21.4 %)	1.0
Splenomegaly	13/54 (24.1 %)	6/40 (15.0 %)	7/14 (50.0 %)	**0.025**
Heterogeneous liver parenchyma	52/54 (96.3 %)	38/40 (95.0 %)	14/14 (100.0 %)	1.0
Segmental atrophy/hypertrophy	16/54 (29.6 %)	10/40 (25.0 %)	6/14 (42.9 %)	0.38
Hepatic vein dilatation	48/54 (88.9 %)	36/40 (90.0 %)	12/14 (85.7 %)	0.643
Abnormal hepatic vein architecture	38/54 (70.4 %)	26/40 (65.0 %)	11/14 (78.6 %)	0.507
Hyperechogenic lesions	10/54 (18.5 %)	7/40 (17.5 %)	3/14 (21.4 %)	0.708
Surface nodularity	8/54 (14.8 %)	3/40 (7.5 %)	5/14 (35.7 %)	**0.021**
Ascites	7/54 (13.0 %)	1/40 (2.5%)	6/14 (42.9%)	**0.001**
Maximum liver function capacity (μg/kg/h)	355.0 (160.8)	390.0 (162.0)	288.0 (140.0)	**0.007**

Data are presented as median and interquartile range or frequencies (%). Statistically significant results are given in bold letters. ALT, Alanin-Aminotransferase; AST, Aspartat-Aminotransferase; γGT, γ-glutamyl-transferase; kPA, Kilopascal; TE, transient elastography.

### Maximal liver function capacity

**Figure 1:**
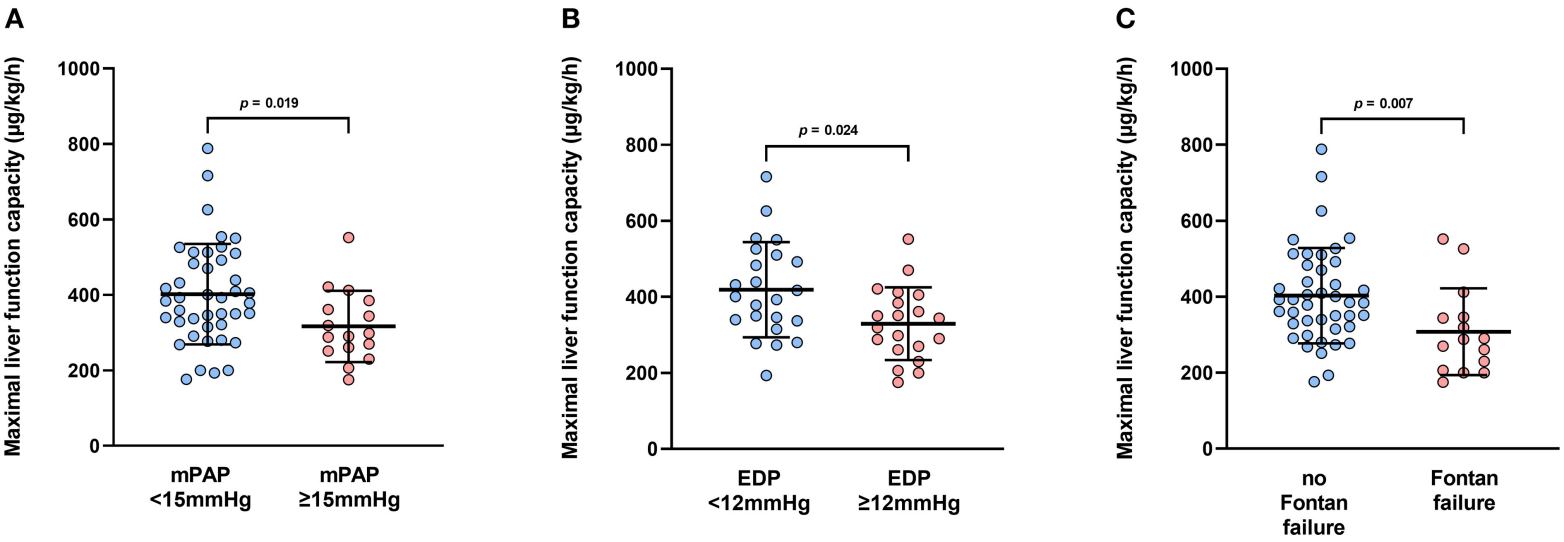
**(A)** Boxplots depict maximal liver function capacity according to mean pulmonary artery pressure (mPAP). mPAP <15 mmHg: n = 42; mPAP <15 mmHg: n = 15. **(B)** Boxplots depict maximal liver function capacity according to systemic ventricular end-diastolic pressure (SVEDP). SVEDP <12 mmHg: n = 23; SVEDP <12 mmHg: n = 19. **(C)** Boxplots depict maximum liver function capacity according to the presence of Fontan failure. Fontan failure; n = 15; no Fontan failure, n = 43.

Median maximum liver function capacity was 355.0 μg/kg^*^h (IQR 160.8), which corresponds to a normal hepatic function (≥ 315 μg/kg^*^h). In 18 patients maximum liver function capacity was moderately impaired (140–314 μg/kg^*^h), while no patient suffered from severe hepatic damage (≤ 139 μg/kg^*^h). No correlation was detected between systolic ventricular function or the extent of AVVI and maximum liver function capacity (*p* = 0.178, r = 0.186 and *p* = 0.873, r = −0.016, respectively). Additionally, no association was found between VO_2_peak and LiMAx® test results (*p* = 0.356, r = 0.133). No correlation was detected between maximal liver function capacity and resting or peak oxygen saturation (*p*_1_ = 0.202, r = 01.7; *p*_2_ = 0.056, r = −0.267). In patients with mPAP ≥ 15 mmHg maximal liver function capacity was significantly reduced compared to patients with mPAP <15 mmHg [294.0 μg/kg^*^h (IQR 126.0) vs. 388.5 μg/kg^*^h (IQR 177.0), *p* = 0.019, Figure 1A]. An SVEDP ≥ 12 mmHg was also associated with decreased maximum liver function capacity [331.5 μg/kg^*^h (IQR 136.5) vs. 401.0 μg/kg^*^h (IQR 173.0), *p* = 0.029; Figure 1B]. Finally, in patients with late Fontan failure, maximal liver function capacity was significantly impaired as compared to patients without evidence of Fontan failure [288.0 μg/kg/^*^h (IQR 140.0) vs. 390.0 μg/kg^*^h (IQR 162.0), *p* = 0.007; Figure 1C].

## Discussion

This is the first study to describe the association between Fontan hemodynamics, Fontan failure and maximum liver function capacity assessed by LiMAx®. Briefly, no correlation was detected between echocardiographic parameters or CPET results and metabolic liver function. Additionally, hemodynamic parameters such as CI and PVRi also showed no association with results from the LiMAx® test. However, maximum liver function capacity was significantly decreased in patients with increased mPAP (≥15 mmHg), and those with increased SVEDP (≥12 mmHg) as well as in patients with Fontan failure. In these patients, hepatic functional impairment was graded as moderate whereas no severe hepatic failure was detected.

The LiMAx® test was introduced to accurately quantify metabolic liver function based on the hepatocyte-specific cytochrome P4501A2 (CYP1A2) system and was evaluated in several clinical settings (17–19). We previously described the missing correlation between maximum liver function capacity and results from other diagnostic modalities such as laboratory parameters, TE and hepatic ultrasound (11). The major finding of our previous study was that metabolic liver function was preserved in the majority of adult Fontan patients even in those with clear evidence of advanced FALD.

Due to the non-physiological hemodynamics, failure of the Fontan circulation is inevitable in the long term (20–22). Since therapeutic strategies are limited, cardiac transplantation remains the only viable end-stage treatment option but is associated with considerable mortality and morbidity (23). Additionally, guidelines for the timing of cardiac transplantation are missing and delayed listing may result in progressing secondary end-organ damage, which significantly contributes to post-transplant mortality (8, 9). Fontan-associated liver disease (FALD) is the most frequent end-organ dysfunction and encompasses all abnormalities in both liver structure and function with the end-stage being severe liver cirrhosis or hepatocellular carcinoma (24, 25). The indication for a combined heart and liver transplantation in failing Fontan patients is currently subject of ongoing debate. The decision whether a patient may benefit from single or multi-organ transplantation is challenging due to the lack of sound data to support or refute any given approach. Whereas successful isolated heart transplantation has been reported in the presence of hepatic cirrhosis (26), feasibility of combined heart and liver transplantation has also been demonstrated (27, 28). The most commonly encountered scenario in Fontan patients considered for cardiac transplantation is the inevitable presence of some degree of liver fibrosis with most patients demonstrating ‘cirrhotic' alterations on imaging. However, it has been shown that cirrhosis on biopsy is less common and often does not correlate with imaging modalities such as ultrasound, magnetic resonance imaging or computed tomography (29). These discrepancies between imaging and biopsy findings complicate the interpretation and classification of FALD and its clinical significance for therapeutic decision making.

The LiMAx® test may represent a valuable complementary diagnostic modality in the hepatic assessment of Fontan patients and provides a reproducible quantitative measurement of hepatocyte function. Since a deterioration of maximum liver function capacity seems to occur relatively late during the disease course, when a significant impairment of Fontan hemodynamics and Fontan failure is already evident, its occurrence may prove as a valuable indicator for the requirement of a timely evaluation for cardiac transplantation. In our cohort, maximum liver function capacity was moderately reduced in patients who received cardiac transplantation [247.0 μg/kg^*^h (IQR 148.8) vs. 369.5 μg/kg^*^h (IQR 182.5), *p* = 0.029), however, none of these patients fulfilled the criteria for a combined heart and liver transplantation such as hepatocellular carcinoma or severe liver cirrhosis. In patients who survived cardiac transplantation, improvements in morphological and laboratory FALD parameters were detected. This observation has also been reported by other institutions and might underline the remarkable hepatic potential for regeneration (11, 30, 31). Therefore, patients with mild to moderate impairment of metabolic liver function might be appropriate candidates for isolated cardiac transplantation, whereas a severely impaired maximum liver function capacity may indicate the necessity of a combined heart and liver transplantation. Since both, FALD and Fontan failure, are characterized by a slow progress and are often clinically disguised by patient‘s adaptation to their chronically reduced output state and clinical deterioration, it seems advisable to perform repeated measurement of maximum liver function capacity during long-term follow-up, however, based on the currently available data, no precise intervals can be recommended. In patients with severe hemodynamic and hepatic impairment a yearly evaluation might be required followed by the consultation of an experienced hepatologist.

However, further well-conducted research efforts are warranted to address these questions, including additional studies, which compare maximum liver function capacity before and after cardiac transplantation as well as explore correlations of hepatic metabolic function with histologic findings of liver biopsies.

## Limitations

This study has several limitations. Since this is a cross-sectional single center study with a comparably small patient cohort, future multi-institutional studies are necessary to evaluate metabolic liver function in larger patient cohorts. Additionally, the longitudinal relationship between Fontan hemodynamics and hepatic function was not addressed by this study. Since the parameters SVEDP and PAP are included in the calculation of the Fontan failure score, the association between LiMAx and Fontan failure might be confounded. However, considering that SVEDP and PAP are only 2 of 15 parameters used for score calculations, the confounding effect seems negligible. Additionally, all of the 15 Fontan failure patients fulfill the clinical consensus definition criteria of Fontan failure (14). Parts of the data of our current study cohort (n = 38/58, 65%) have previously been published in a study with a different scope focusing on morphologic hepatic assessment (11).

## Conclusions

We herein demonstrate that maximum liver function capacity measured by the LiMAx® test is impaired in patients with impaired Fontan hemodynamics and Fontan failure. Hence, the LiMAx® test represents a valuable complementary diagnostic modality for FALD and might be useful in evaluating the indication for combined heart and liver transplantation.

## Data availability statement

The raw data supporting the conclusions of this article will be made available by the authors, without undue reservation.

## Ethics statement

The studies involving human participants were reviewed and approved by Ethikkommission der Charité, Berlin. The patients/participants provided their written informed consent to participate in this study.

## Author contributions

Conceptualization and formal analysis: AS, PK, and SO. Data collection: AS, NJ, MP, and PK. Investigation: AS, PK, FD, H-PM, TM, and HS. Supervision: FB and FT. Writing original draft: AS. Writing review and editing: PK and SO. All authors contributed to the article and approved the submitted version.

## Funding

Parts of the study were funded by Kinderherzen e.V., who also finance the open access publication of the manuscript.

## Conflict of interest

The authors declare that the research was conducted in the absence of any commercial or financial relationships that could be construed as a potential conflict of interest.

## Publisher's note

All claims expressed in this article are solely those of the authors and do not necessarily represent those of their affiliated organizations, or those of the publisher, the editors and the reviewers. Any product that may be evaluated in this article, or claim that may be made by its manufacturer, is not guaranteed or endorsed by the publisher.
